# GTDB-Tk v2: memory friendly classification with the genome taxonomy database

**DOI:** 10.1093/bioinformatics/btac672

**Published:** 2022-10-11

**Authors:** Pierre-Alain Chaumeil, Aaron J Mussig, Philip Hugenholtz, Donovan H Parks

**Affiliations:** Australian Centre for Ecogenomics, School of Chemistry and Molecular Biosciences, The University of Queensland, St Lucia, QLD 4072, Australia; Research Computing Center, The University of Queensland, St Lucia, QLD 4072, Australia; Australian Centre for Ecogenomics, School of Chemistry and Molecular Biosciences, The University of Queensland, St Lucia, QLD 4072, Australia; Australian Centre for Ecogenomics, School of Chemistry and Molecular Biosciences, The University of Queensland, St Lucia, QLD 4072, Australia; Australian Centre for Ecogenomics, School of Chemistry and Molecular Biosciences, The University of Queensland, St Lucia, QLD 4072, Australia

## Abstract

**Summary:**

The Genome Taxonomy Database (GTDB) and associated taxonomic classification toolkit (GTDB-Tk) have been widely adopted by the microbiology community. However, the growing size of the GTDB bacterial reference tree has resulted in GTDB-Tk requiring substantial amounts of memory (∼320 GB) which limits its adoption and ease of use. Here, we present an update to GTDB-Tk that uses a divide-and-conquer approach where user genomes are initially placed into a bacterial reference tree with family-level representatives followed by placement into an appropriate class-level subtree comprising species representatives. This substantially reduces the memory requirements of GTDB-Tk while having minimal impact on classification.

**Availability and implementation:**

GTDB-Tk is implemented in Python and licenced under the GNU General Public Licence v3.0. Source code and documentation are available at: https://github.com/ecogenomics/gtdbtk.

**Supplementary information:**

Supplementary data are available at *Bioinformatics* online.

## 1 Introduction

The Genome Taxonomy Database (GTDB) and associated taxonomic classification toolkit (GTDB-Tk) has been used to assign taxonomic classifications to tens of thousands of bacterial and archaeal isolate genomes and metagenome-assemble genomes recovered from environmental and human-associated samples (Almeida *et al.*, 2021; Chaumeil *et al.*, 2019; Nayfach *et al.*, 2021). These classifications are consistent with the GTDB framework and based on the same relative evolutionary divergence (RED) and average nucleotide identity (ANI) criteria for circumscribing taxa (Parks *et al.*, 2020, 2022). A primary step in assigning classifications is placing genomes into the GTDB bacterial or archaeal reference trees using the maximum likelihood (ML) placement tool pplacer (Matsen *et al.* 2010). Unfortunately, ML placement with pplacer is a memory-intensive operation requiring ∼320 GB of RAM when using the GTDB R07-RS207 bacterial reference tree comprised of 62 291 genomes. Adding to this challenge is the lack of favourable alternatives to pplacer as the EPA-ng ML placement method requires more memory than pplacer and distance-based methods such as APPLES-2 have inferior performance (Balaban *et al.*, 2022; Barbera *et al.*, 2019; Koning *et al.*, 2021). The GTDB bacterial reference tree has been growing rapidly in size with each GTDB release and is expected to grow by upwards of 30% per year for the next few years (Parks *et al.*, 2022). Unfortunately, the size of this tree results in the memory requirements of GTDB-Tk being impractical. Here, we show that the memory requirements of GTDB-Tk are reduced by dividing the GTDB bacterial reference tree into class-level subtrees and demonstrate that taxonomic classifications are largely unimpacted by this change.

## 2 Materials and methods

GTDB-Tk v2 divides the GTDB bacterial reference tree into class-level subtrees to reduce memory requirements. Placement of a genome with pplacer now consists of two steps. First, a genome is placed into a backbone tree consisting of a single genome representative for each family (see Supplementary Methods). If the genome is assigned to a class within this backbone tree, it is then placed into a class-level subtree to obtain a more refined placement for the genome.

The class-level subtrees were constructed in a greedy manner with the maximum size of a class-level subtree being set based on the number of species representatives in the largest class (*Gammaproteobacteria* with 9582 genomes in GTDB R07-RS207). Each class-level subtree was formed by selecting the largest class in the reference tree and traversing towards the root until the subtree contained at most 10 540 genomes (10% more than the *Gammaproteobacteria*). This subtree was then pruned from the reference tree and the procedure repeated until all classes were assigned to a class-level subtree. For GTDB R07-RS207, this resulted in seven class-level trees. Each class-level subtree was then expanded to contain a single genome from each phylum in order to allow query genomes to be placed as the most basal member of a class.

Final taxonomic classifications use the same RED and ANI criterion as GTDB-Tk v1 (Chaumeil *et al.*, 2020) with the following additional rules:

1. A genome not placed into a class-level subtree is assigned the classification determined in the backbone tree.
2. A genome placed into a class-level subtree and assigned to a phylum belonging to one of the classes contained in the subtree is assigned the classification determined in the subtree.
3. Otherwise, the genome is classified by taking the lowest common ancestor between the backbone and class-level subtree.

## 3 Results

Here, we demonstrate that the taxonomic classifications produced by the divide-and-conquer approach implemented in GTDB-Tk v2 are nearly equivalent to those produced by GTDB v1 while providing a substantial reduction in required memory.

### 3.1 Similarity of classifications on diverse sets of genomes

The concordance between GTDB-Tk v1 and v2 classifications was first assessed using 16 710 bacterial genomes from the GEMs dataset (Nayfach *et al.*, 2021) that represent novel taxa relative to GTDB R07-RS207 (Table 1). Only 12 genomes (0.07%) did not have identical classifications between GTDB-Tk v1 and the divide-and-conquer approach used in GTDB-Tk v2 (Supplementary Table S1). The majority of incongruence was due to genomes being over- (six genomes) or under-classified (four genomes) by a single taxonomic rank. Only two genomes had conflicting taxonomic assignments, and these were both relatively poor-quality genomes assigned as new classes in alternative phyla (Supplementary Table S1).

**Table 1.** Novelty of GEM genomes relative to GTDB R07-RS207 based on GTDB-Tk v1 classifications

		GTDB-Tk v2 classifications relative to GTDB-Tk v1 classifications			
Taxon novelty	No. genomes	Congruent	Conflict^a^	Underclassified^b^	Overclassified^c^
Novel phylum	3	2	0	0	1
Novel class	42	36	2	2	2
Novel order	144	143	0	0	1
Novel family	543	540	0	1	2
Novel genus	3222	3221	0	1	0
Novel species	12 756	12 756	0	0	0

*Note*: GTDB-Tk v2 predicts a different taxon (a), or less (b) or more (c) resolved classifications than GTDB-Tk v1 (see Supplementary Table S1).

GTDB-Tk v1 and v2 classifications were further evaluated by dereplicating the ∼60 000 genomes introduced in GTDB R07-RS207 to 23 548 genomes by randomly selecting a single genome per species. These 23 548 genomes were then classified using the GTDB-Tk R06-RS202 reference package to further evaluate classifications on genomes with varying degrees of taxonomic novelty and to ensure results were robust with different GTDB reference packages (Supplementary Table S2). Only 13 genomes (0.06%) had different GTDB-Tk v1 and GTDB-Tk v2 classifications with 5 having conflicting assignments, 5 being overclassified and 3 being underclassified (Supplementary Table S3).

### 3.2 Reduced memory requirements

The divide-and-conquer approach implemented in GTDB-Tk v2 reduced the maximum memory requirements from ∼320 GB to <55 GB when run with the GTDB R07-RS207 reference trees. GTDB-Tk v2 also ran 22–35% faster when processing 1000 genomes with 1–64 CPUs (Supplementary Fig. S1A) and was >40% faster when processing 5000 genomes using 32 CPUs (Supplementary Fig. S1B).

## 4 Summary

GTDB-Tk v2 requires only a sixth of the memory of GTDB-Tk v1 while providing almost identical classifications. More importantly, the divide-and-conquer approach used in GTDB-Tk v2 allows memory requirements to be controlled by tailoring the size of the largest subtree. This ensures GTDB-Tk can continue to be used on readily available computing hardware even as the size of the GTDB bacterial reference tree increases.

## Supplementary Material

btac672_Supplementary_Data
