# Supplementary material for: GTDB-Tk v2: memory friendly classification with the genome taxonomy database
Source: Bioinformatics. 2022 Oct 11;38(23):5315–6. doi: 10.1093/bioinformatics/btac672 (PMC9710552; doi:10.1093/bioinformatics/btac672)
Supplement: btac672_Supplementary_Data [file btac672_supplementary_data.docx]

**Supplementary Material**

**GTDB-Tk v2: memory friendly classification with the Genome Taxonomy Database**

Pierre-Alain Chaumeil, Aaron J. Mussig, Philip Hugenholtz, Donovan H. Parks

**Supplementary Methods**

The backbone tree used in GTBD-Tk v2 consists of a single genome for each family. Assembly quality and NCBI metadata was used to establish the genome selected to represent each family. Specifically, each genome was assigned a score as follows:

- CheckM completeness estimate – 5 $\times$ CheckM contamination estimate
- -5 $\times$ number of contigs / 100
- -5 $\times$ number of ambiguous bases / 10000
- -5$\times$ percentage of gaps in GTDB multiple sequence alignment
- -100 if assembly is from a MAG or SAG

The highest scoring genome in each GTDB family was selected for use in the GTDB-Tk v2 backbone tree.

**Supp. Table 1.** Incongruent GTDB-Tk v1 and v2 classifications across 16,710 GEM MAGs.

| **GEMs ID** | **CheckM completeness (%)** | **CheckM contamination (%)** | **GTDB-Tk v1 classification** | **GTDB-Tk v2 classification** | **Type of Change** |
| --- | --- | --- | --- | --- | --- |
| 3300017989_37 | 75.27 | 0 | d__Bacteria; p__4572-55 | d__Bacteria; p__Gemmatimonadota | Conflict |
| 3300024433_39 | 62.46 | 0 | d__Bacteria; p__TA06_A | d__Bacteria; p__Gemmatimonadota | Conflict |
| 3300025546_16 | 96.27 | 0 | d__Bacteria | d__Bacteria; p__Aerophobota | Overclassified |
| 3300005529_81 | 70.3 | 0 | d__Bacteria; p__Chloroflexota | d__Bacteria; p__Chloroflexota; c__Ktedonobacteria | Overclassified |
| 3300013133_46 | 89.51 | 1.12 | d__Bacteria; p__Firestonebacteria | d__Bacteria; p__Firestonebacteria; c__D2-FULL-39-29 | Overclassified |
| 3300025847_116 | 64.12 | 0 | d__Bacteria; p__Caldisericota; c__Caldisericia; o__JAAYUI01 | d__Bacteria; p__Caldisericota; c__Caldisericia; o__JAAYUI01; f__JAAYUI01 | Overclassified |
| 3300025856_28 | 56.8 | 1.23 | d__Bacteria; p__OLB16; c__OLB16 | d__Bacteria; p__OLB16; c__OLB16; o__SURF-12 | Overclassified |
| 3300027863_103 | 83.62 | 3.45 | d__Bacteria; p__Actinobacteriota; c__Thermoleophilia; o__UBA2241 | d__Bacteria; p__Actinobacteriota; c__Thermoleophilia; o__UBA2241; f__UBA2241 | Overclassified |
| 3300000227_24 | 93.28 | 0.84 | d__Bacteria; p__Desulfobacterota_D | d__Bacteria | Underclassified |
| 3300006083_11 | 81.72 | 0.81 | d__Bacteria; p__Aquificota; c__Desulfurobacteriia; o__Desulfurobacteriales; f__Desulfurobacteriaceae | d__Bacteria; p__Aquificota; c__Desulfurobacteriia; o__Desulfurobacteriales | Underclassified |
| 3300026127_6 | 81.7 | 4.46 | d__Bacteria; p__Desulfobacterota_D; c__UBA1144; o__RKRQ01 | d__Bacteria; p__Desulfobacterota_D; c__UBA1144 | Underclassified |
| 3300026488_14 | 68.64 | 0 | d__Bacteria; p__Muirbacteria | d__Bacteria | Underclassified |

**Supp. Table 2.** Novelty of 23,548 GTDB R07-RS207 genomes relative to R06-RS202 based on GTDB-Tk v1 classifications.

|  | **No. genomes** | **GTDB-Tk v2 classifications relative to GTDB-Tk v1 classifications** |
| --- | --- | --- |
| Novel phylum | 3 | 3 congruent |
| Novel class | 32 | 28 congruent; 3 conflict; 1 underclassified |
| Novel order | 199 | 194 congruent; 2 conflict; 1 underclassified; 2 overclassified |
| Novel family | 580 | 577 congruent; 1 underclassified; 2 overclassified |
| Novel genus | 3,393 | 3,392 congruent; 1 overclassified |
| Novel species | 12,616 | 12,616 congruent |
| Known Species | 6,725 | 6,725 congruent |

**Supp. Table 3.** Incongruent GTDB-Tk v1 and v2 classifications across 23,548 MAGs introduced in GTDB R07-RS207.

| **GEMs ID** | **CheckM completeness (%)** | **CheckM contamination (%)** | **GTDB-Tk v1 classification** | **GTDB-Tk v2 classification** | **Type of Change** |
| --- | --- | --- | --- | --- | --- |
| GB_GCA_018818825.1 | 97.75 | 1.12 | d__Bacteria; p__UBA9089; c__UBA9089 | d__Bacteria; p__UBA9089; c__CG2-30-40-21 | Conflict |
| GB_GCA_016209155.1 | 86.41 | 4.03 | d__Bacteria; p__Aerophobota | d__Bacteria; p__UBP18; c__UBA7526 | Conflict |
| GB_GCA_016926495.1 | 76.57 | 1.68 | d__Bacteria; p__Bdellovibrionota; c__FAC87 | d__Bacteria; p__Bdellovibrionota; c__YA12-FULL-61-11 | Conflict |
| GB_GCA_016190245.1 | 75.18 | 0.84 | d__Bacteria; p__J088 | d__Bacteria; p__SpSt-318 | Conflict |
| GB_GCA_016783345.1 | 63.18 | 0 | d__Bacteria; p__NPL-UPA2 | d__Bacteria; p__CAIJMQ01 | Conflict |
| GB_GCA_017861215.1 | 81.27 | 0.81 | d__Bacteria; p__Desulfobacterota_F; c__Desulfuromonadia; o__Geobacterales; f__Geobacteraceae | d__Bacteria; p__Desulfobacterota_F; c__Desulfuromonadia; o__Geobacterales; f__Geobacteraceae; g__VEOV01 | Overclassified |
| GB_GCA_018829865.1 | 70.89 | 2.97 | d__Bacteria; p__Patescibacteria; c__Gracilibacteria; o__UBA1369 | d__Bacteria; p__Patescibacteria; c__Gracilibacteria; o__UBA1369; f__PNNO01 | Overclassified |
| GB_GCA_016927475.1 | 90.37 | 2.56 | d__Bacteria; p__Acidobacteriota; c__Aminicenantia; o__Aminicenantales | d__Bacteria; p__Acidobacteriota; c__Aminicenantia; o__Aminicenantales; f__Aminicenantaceae | Overclassified |
| GB_GCA_015231965.1 | 95.66 | 0 | d__Bacteria; p__Proteobacteria; c__Magnetococcia | d__Bacteria; p__Proteobacteria; c__Magnetococcia; o__Magnetococcales | Overclassified |
| GB_GCA_016212085.1 | 99.49 | 0 | d__Bacteria; p__Nitrospirota; c__Nitrospiria | d__Bacteria; p__Nitrospirota; c__Nitrospiria; o__SBBL01 | Overclassified |
| GB_GCA_016180645.1 | 89.33 | 3.36 | d__Bacteria; p__SpSt-318 | d__Bacteria | Underclassified |
| GB_GCA_015231815.1 | 79.81 | 1.71 | d__Bacteria; p__Nitrospinota; c__UBA7883 | d__Bacteria | Underclassified |
| GB_GCA_015233785.1 | 61.19 | 2.1 | d__Bacteria; p__Proteobacteria; c__Magnetococcia; o__Magnetococcales | d__Bacteria; p__Proteobacteria; c__Magnetococcia | Underclassified |


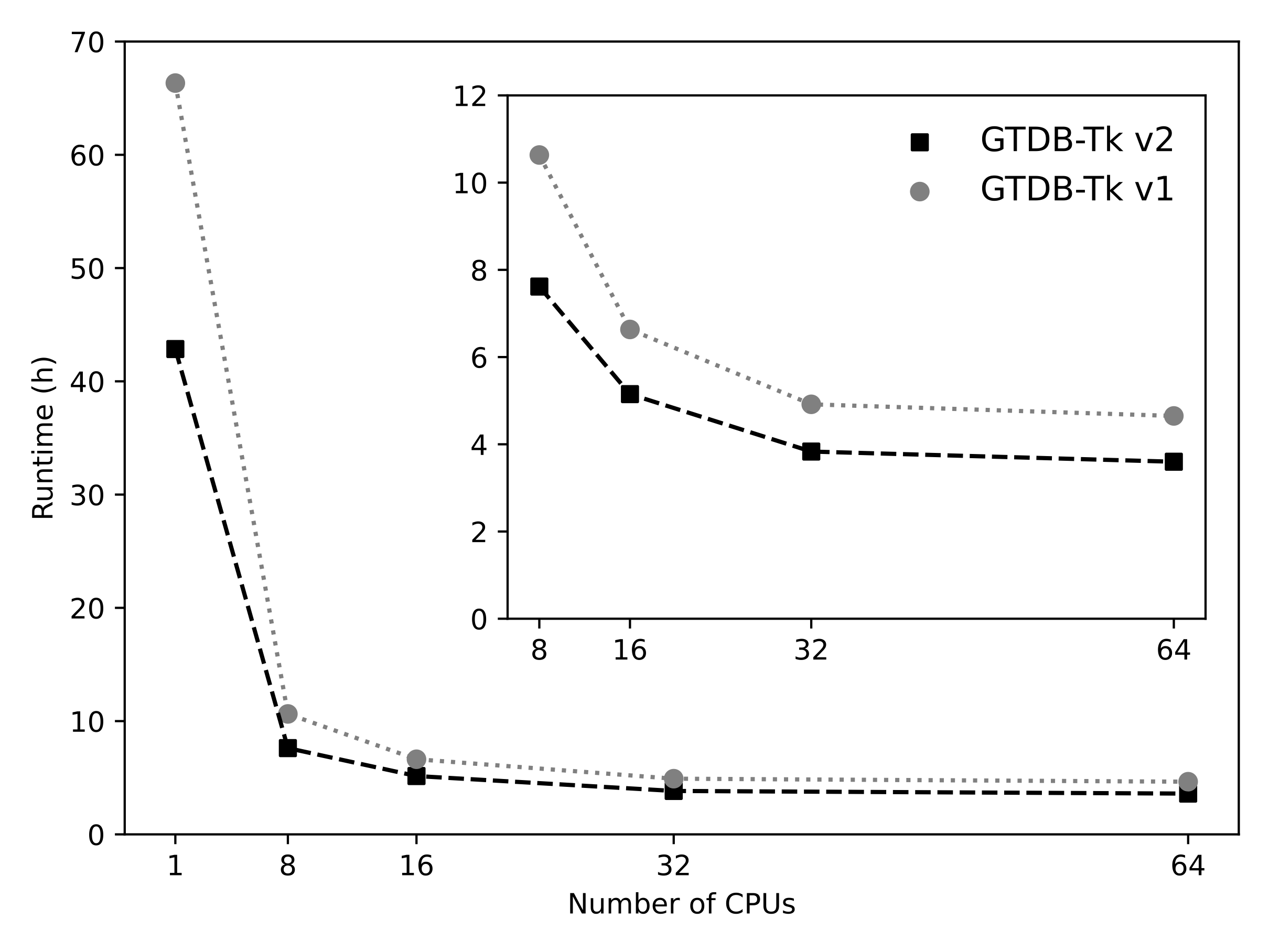

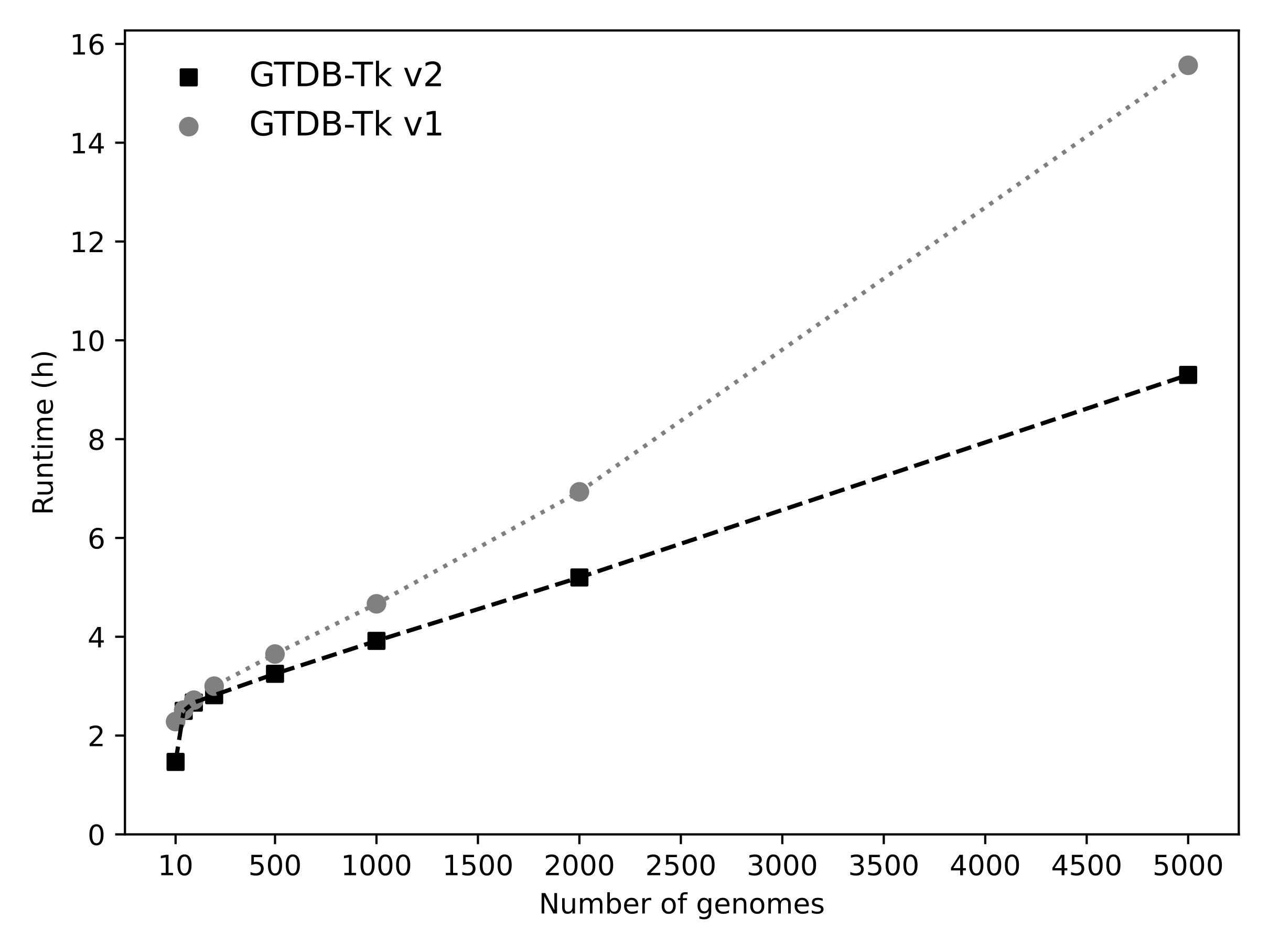


a

b

**Supp. Figure 1**. Reduced processing time of GTDB-Tk v2 compared to GTDB-Tk v1. (**a**; **left**) Processing time (log scale) for 1,000 randomly selected GEM MAGs for increasing numbers of CPUs. (**b**; **right**) Processing time with 32 CPUs on increasing numbers of randomly selected GEM MAGs. Tests were run on a machine with 4 AMD EPYC 7402 24-Core Processor and 512 GB of RAM.
